# The Effect of Anti-browning Agent Activated Carbon and Polyvinyl Pyrrolidone on the Rooting of Embryo Seedlings of “FengDan” and Its Transcriptome Analysis

**DOI:** 10.3389/fpls.2022.832619

**Published:** 2022-03-21

**Authors:** Xia Chen, Chao Yu, Jingtao Nie, Hongmin Yang, Wen Ji, Gangwei Xu, Huijun Zhu, Songheng Jin, Xiangtao Zhu

**Affiliations:** ^1^College of Jiyang, Zhejiang A&F University, Zhuji, China; ^2^College of Horticulture Science, Zhejiang A&F University, Hangzhou, China; ^3^Educational Technology Center of Zhejiang Province, Hangzhou, China; ^4^Zhejiang Provincial Key Laboratory of Germplasm Innovation and Utilization for Garden Plants, Zhejiang A&F University, Hangzhou, China

**Keywords:** *Paeonia suffruticosa* Andr., embryo seedling, anti-browning agent, root, transcriptome

## Abstract

Peony is an excellent ornamental, medicinal, and oily plant. Its traditional seed propagation methods have the disadvantages of low propagation coefficient, long seedling cycle, and low seedling emergence rate, which severely restrict the supply of seedlings for the peony industry. Efficient tissue culture technology is an important basis for accelerating its breeding and reproduction, and *in vitro* seed embryo culturing into seedlings can also effectively avoid the above problems. However, the browning phenomenon caused by man-made damage in the process of seed embryo stripping leads to problems such as low induction rate and difficulty in rooting, and the relationship between anti-browning agents and seed embryo root formation is still unclear. This study intends to improve the induction rate of peony seedlings by using different anti-browning agents and different combinations and to clarify the relationship between anti-browning agents and seedling rooting using transcriptome sequencing methods. The results show that both anti-browning agents, activated carbon (AC) and polyvinyl pyrrolidone (PVP), can increase the germination rate of seed embryos. Testing with 0.9 g/L of AC showed excellent performance of peony rooting rate and seedling growth, but only AC and the combination of AC and PVP can further promote rooting development. Through transcriptome analysis, we found that the AC vs. control check (CK), AC vs. PVP, and PVP vs. AC and PVP groups have significantly more differentially expressed genes than the AC vs. AC and PVP groups. Pathway enrichment analysis shows that “phenylpropanoid biosynthesis”/“cutin, suberin, and wax biosynthesis” is significantly enriched in these groups, while the AC vs. AC and PVP groups are mainly enriched in “cytochrome P450,” indicating that AC may promote the further development of roots into seedlings by stimulating “phenylpropanoid biosynthesis” and biosynthesis of stratum cutin and suberin. This study can lay the foundation for understanding the potential molecular mechanism of the anti-browning agent promoting the rooting of seed embryo seedlings and also provide a theoretical basis for perfecting the construction of the peony tissue culture and rapid propagation system.

## Introduction

Peony (*Paeonia suffruticosa* Andr.) has excellent garden ornamental value and medicinal value (Li et al., 2015). The current peony propagation method is mainly seed propagation, attached to grafting, ramets, cuttings, and other asexual propagation (Yang, 2009; Yang et al., 2015; Zhu et al., 2018), and its low reproduction coefficient, long seedling cycle, low seedling emergence, and uneven quality (Wang, 2016) have made the current supply of seedlings a bottleneck, restricting the development of the peony industry. As a new method of plant propagation, tissue culture technology has been widely popularized. The advantage is that it can multiply a large number of fine varieties in a short period of time, breaking the limitations of traditional breeding techniques and can use genetic transformation technology to cultivate new varieties of peony (Li et al., 2018). Therefore, solving the problems in the conventional breeding process of peony through tissue culture technology will be an inevitable trend for the development of peony to commercialization and industrialization, and it is also an effective measure to accelerate its reproduction and breeding (Xiu et al., 2017). This study on peony tissue culture technology mainly focuses on callus induction, suitable growth regulator ratio, explant selection, transplantation and domestication, etc. (Li et al., 2008; Cheng et al., 2019; Ma X. L. et al., 2019; Huang, 2020; Shi et al., 2021; Zhang et al., 2021). Although certain progress has been made, there are still problems in the process of peony tissue culture, such as serious pollution, prone to browning and vitrification in the subculture, difficulty in rooting, and low survival rate of transplanting (Jia and Liu, 2014), which result in the failure to establish an efficient and stable system and are unable to provide assistance to the actual production.

Studies have shown that using seed embryos as explants for *in vitro* culture can not only effectively avoid the above problems but also enable the embryos to germinate into seedlings quickly and advance the germination time, and it is of great significance to save hybrid embryo abortion, relieve seed dormancy, advance the breeding cycle, and increase the germination rate (Yu et al., 2013; He, 2019; Lian et al., 2020). The seed embryo culture technology has been successful in many crops and horticultural plants (Li et al., 2020; Ongagna et al., 2020; Bhatia et al., 2021), and research has also been done in the rapid propagation of peony. For example, Liu et al. (2011) used the peony variety “FengDan” as the material to explore the rapid growth technology of mature embryos through the combination of different hormones and basic media and has found that adding 1.5 mg/L of 6-BA and 1.0 mg/L of NAA to 1/2 Murashige and Skoog (MS) medium had the best effect. Nevertheless, this technology still has many unresolved problems such as low induction rate, poor growth, and development of test-tube plantlets, difficulty in rooting, and low survival rate after transplanting. Among them, the browning phenomenon caused by artificial damage during the process of stripping embryos leads to a low induction rate and difficulty in rooting, which are important factors that restrict and affect the rapid growth of seedlings of peony embryos. In tissue culture, browning is caused by the formation of quinones by phenolic acids under the catalysis of a variety of oxidases after plant materials are injured. Quinones produce colored substances through polymerization to cause tissues to become brown, and this brown substance will gradually spread into the culture medium, poisoning the entire tissue (Li et al., 2008). In plants, phenolic substances are closely related to the metabolism of auxin (IAA) (Fu, 2012), which seriously affects the origin and development of plant adventitious roots. Shang et al. (2020) studied the effects of phenolic substances of different types and concentrations on adventitious root formation of peony test-tube seedlings by adding phenolic substances in the rooting medium and have found that exogenous phenolic substances had a higher rooting rate and rooting index on test-tube seedlings than the control. In addition, endogenous phenols can react with IAA under the action of polyphenol oxidase, and the product has auxin activity and can also promote root formation. To a certain extent, this reveals the relationship between endogenous phenolic substance metabolism and root formation during the rooting process of peony test-tube plantlets. Paeonia is a plant species with a high content of phenolic substances, and its body contains a large amount of phenolic acids (Zhang and Fan, 2008); it can be seen that, in the rapid seedling formation technology of peony embryo culture, preventing the oxidative browning of phenolic acids in seed embryos caused by artificial damage is crucial to promoting the rooting of seed embryo seedlings. At present, the main method to prevent browning in tissue culture is to add anti-browning agents, of which activated carbon (AC), polyvinyl pyrrolidone (PVP), vitamin C, sodium thiosulfate, cysteine and ethylene synthesis inhibitor of aminoethoxyvinylglycine (AVG), silver nitrate, and silver thiosulfate (Li et al., 2008), are commonly used, among which, AC and PVP are the most widely used and have better effects. However, the research on the relationship between anti-browning agents and root formation of peony embryo seedlings is still blank.

Therefore, we studied the effects of two browning inhibitors, namely, AC and PVP, and their combination on the browning control during embryo culture of the peony variety “FengDan” and the effect on the rooting of seedling embryos, in order to improve the induction rate of peony embryo rapid seedling; at the same time, we conducted a transcriptome comparative analysis of the root and leaf mixed tissues of different treatments of peony embryo seedlings to understand the potential molecular mechanism of the anti-browning agent to promote the rooting of seedlings, laying a foundation for further research on the rooting mechanism of peony test-tube seedlings and providing a theoretical basis for the construction of the peony rapid propagation system.

## Materials and Methods

### Plant Materials and Culture Conditions

The peony variety “FengDan” was used as the test material and was planted in the Peony Experimental Base of Jiyang College, Zhejiang Agriculture and Forestry University. Fresh seeds with good growth conditions were picked 120 days after flowering as the source of embryonic explants. The seeds were peeled out of the pods for sterilization, washed with detergent, and rinsed under running water for 10 min. They were transferred to the ultra-clean workbench, soaked in 2% of NaClO solution for 20 min, rinsed with sterile water for 3–5 times, sterilized with 75% of alcohol for 10–30 s, rinsed with sterile water for 3–5 times, then placed on a sterile filter paper to absorb the water, carefully peeled off the seed embryo with a scalpel and tweezers, and inoculated in the MS medium where the seed embryo germinates. The composition of different treatment media is shown in Table 1. The pH value was 5.8. After inoculation, the seeds were cultured in the dark for 30 days to observe the growth status of seed embryos, and the germination rates of *in vitro* embryos of “FengDan” seeds in different treatments were counted and then switched to light for 17 h day^–1^ with the light intensity of 30–50 μmol m^–2^ s^–1^, using an LED (70% red light + 30% blue light) at a temperature of 24(±2)°C to observe the rooting situation. A total of 30 biological replicates were prepared for each group, with 4 seed embryos in each replicate. SPSS 20.0 software (IBM, Untied States) was used for statistical analysis.

**TABLE 1:** Composition of media for different treatment combinations.

Group	Basal medium	PVP (g⋅L^–1^)	AC(g⋅L^–1^)
CK		–	–
1P		1.5	–
2P		2.0	–
3P		2.5	–
4P		3.0	–
1A	MS 4.74 g/L	–	0.3
2A	Agar 8.5 g/L	–	0.6
3A	Suc 30.0 g/L	–	0.9
4A	IAA 1.4 mg/L	–	2.0
1P-A		1.5	0.9
2P-A		2.0	0.9
3P-A		2.5	0.9
4P-A		3.0	0.9

*1–4P represents the groups added with different concentrations of PVP; 1–4A represents the groups added with different concentrations of AC; and 1–4P-A represents the groups added with different concentrations of AC and PVP combinations.*

To further analyze the effect mechanism of different types and combinations of anti-browning agents on seed embryo seedling rooting, among different treatment groups, the samples were collected from the group with better germination rates and obvious rooting differences. Each sample is composed of 10 mixed leaves and roots of separate seed embryos. Three biological replicates were paired for each sample, immediately frozen in liquid nitrogen, and stored at −80°C.

### mRNA Isolation and cDNA Library Construction and Transcriptome Sequencing

Total RNA was isolated using a TRIzol total RNA extraction kit (ComWin, Beijing, China), following the instructions of the manufacturer, which yielded >2 μg of total RNA per sample. RNA quality was examined using 0.8% agarose gel electrophoresis and spectrophotometer. High-quality RNA with a 260/280 absorbance ratio of 1.8–2.2 was used for library construction and sequencing. Illumina HiSeq library construction was performed according to the instructions of the manufacturer’ (Illumina, United States). Oligo-dT primers were used to transverse mRNA to obtain cDNA (APExBIO, Cat. No. K1159). cDNA was amplified for the synthesis of the second chain of cDNA. cDNA products were purified using magnetic beads. After library construction, library fragments were enriched by PCR amplification and selected according to a fragment size of 350–550 bp. The library was quality-assessed using an Agilent 2100 Bioanalyzer (Agilent, United States). The library was sequenced using the Illumina NovaSeq 6000 sequencing platform (Paired end150) to generate raw reads (APExBIO Co. Ltd., China).

### *De novo* Transcriptome Assemblies

The raw reads of 12 individual assemblies were filtered to remove low-quality reads and reads containing adapter sequence before performing the assembly using the TrimGalore (version 0.6.4) software.^1^ *De novo* transcriptome assembly was accomplished using the Trinity software (version 2.4.0) (Haas et al., 2013), by which transcripts and unigenes (the longest transcript of a set of transcripts that appear to stem from the same transcription locus) were obtained.

### Quantification for Assembled Transcripts

The assembled transcriptome was used as a reference database, and clean reads were mapped back to the reference transcriptome by using Bowtie (version 1.2.3) (Langmead, 2009), and all assembled unigenes counts were quantified using RSEM (version 1.3.3) (Dewey and Li, 2011). Then, the data were normalized for variation in sequencing depth using the transcripts per million (TPM) method (Patro et al., 2017).

### Functional Annotation of Transcripts

Transcriptome functional annotation was performed using Trinotate pipeline (version 3.2.2) (Bryant et al., 2017). Gene functions of all the assembled unigenes were annotated based on the following databases with a cut-off *E*-value of 1.0 × 10^–5^: Nr (NCBI nonredundant protein sequences), Pfam (protein family), and Swiss-Prot (a manually annotated and reviewed protein sequence database). Gene Ontology (GO) and Kyoto Encyclopedia of Genes and Genomes (KEGG) annotation results were retrieved from eggNOG-mapper^2^ and GhostKOALA.^3^

### Sequence Mapping and Differential Expression Analysis

DEGseq2 R package (version 1.28.1) (Love et al., 2014) was used for differential expression analysis using raw read counts. The *P*-value was adjusted using Benjamini–Hochberg methods as adjusted *P*-value. Adjusted *P*-value < 0.05 and | log2 FC (fold change) | > 1 were defined as the threshold for differentially expressed genes (DEGs). Expression profiles of DEGs were examined by functional and pathway enrichment analysis using GO data and KEGG terms. For GO and KEGG enrichment analysis, we used the cluster Profiler R Package (version 3.16.1) (Yu et al., 2012).

### Real-Time Quantitative PCR Verification Analysis

To verify the reliability of the transcriptome information, 19 DEGs were randomly selected for real-time quantitative PCR (qRT-PCR). The selected gene names and primer information are shown in Table 2. RNA extraction and reverse transcription were performed according to the methods described by Chen et al. (2021). The peony ubiquitin (Wang et al., 2012) gene was used as the housekeeping gene (ubiquitin-F: GACCTATACCAAGCCGAAG and ubiquitin-R: CGTTCCAGCACCACAATC), and the relative expression level of the genes was analyzed by qRT-PCR using the applied biosystem PowerUp SYBR Green kit. Three technical replicates for each sample were analyzed. The reaction system is as follows: 50°C for 2 min, 95°C for 2 min, 1 cycle; 95°C for 15 s, 60°C for 15 s, 72°C for 1 min, 40 cycles; 95°C for 15 s, 60°C for 1 min, 95°C for 15 s, 1 cycle.

**TABLE 2:** Primers used in qRT-PCR.

Gene names	Forward primer (5′ to 3′)	Reverse Primer (5′ to 3′)
*TRINITY_DN1028_c0_g1*	GGAGAGTAGGAAGTGGTGGG	ACCTCCTGCACTTCTTCCTC
*TRINITY_DN11050_c1_g1*	GATGGGTCTAGTCCTGCTCC	GGGGACAAACTCATGAGCAC
*TRINITY_DN1323_c0_g1*	CACATTTGCCAGGCTCAACT	AGTTGGGGTGATGATGGGAG
*TRINITY_DN6_c0_g3*	CCTATGGCGACTACTGGAGG	AACTTCTTCTTCCCTCGCCA
*TRINITY_DN42643_c0_g1*	CGTGTCAGTCTTGTGTGTCG	AGGTAGAGAAAGGCACGGAC
*TRINITY_DN960_c0_g1*	TTGCAGTGGGATTAGGGGTC	CAGCTGACTTCAAGCACCTG
*TRINITY_DN10171_c0_g1*	TTGTGCTGCGGTGAAGAAAA	TATTGCAGCGCTTGGGAATG
*TRINITY_DN10_c0_g1*	TCTCCTTAATCTGGCCCACC	ACCGGCACTTACGATTCCTA
*TRINITY_DN12346_c0_g1*	ATGCAGTTGTTGACGTTGCT	GCTGACGTTACACAAGCCAA
*TRINITY_DN1083_c0_g1*	CTTGCAGACACCCACAACTC	AATTAGGGCTTCGGGGATCC
*TRINITY_DN1166_c1_g1*	GCAGCACTGTATCGAAGGTG	ATGGCTGTAACATGCAACCC
*TRINITY_DN11019_c1_g1*	AGGCTGAATCACCCATGGAA	TCCTGCACCTTGTCCGTTAT
*TRINITY_DN1430_c0_g3*	GGGATGGTCAACTGGGAGAA	CTCCTCTCCTTTCCCTGCAA
*TRINITY_DN1131_c0_g1*	ACCCCAAACCCAGATCGATT	GATTTTCCCAGTGACCGACG
*TRINITY_DN16973_c0_g1*	ATGGTCCTAGCTTGCATGGT	GGCATGGGGTTAGGCTTCTA
*TRINITY_DN1271_c0_g1*	AACCCATCCAACTGACCGAT	ATAAGGTCCAGAGGTTGCCG
*TRINITY_DN11889_c1_g1*	CGGATTGAGGTGGAGCGTAT	CTGGTTATGGTTACGGTGCG
*TRINITY_DN15648_c0_g1*	CGTTCGCATGTACAACCCAA	CGTGGATAGGAAGACGGTCA
*TRINITY_DN10081_c0_g1*	GTGTGTCGTCTATTTGCTCGGTCTC	TCTCTGGCCGATGGATGCCCACC

## Results

### Effects of Different Browning Agents on Germination and Rooting of Peony Embryo Seedlings

**FIGURE 1:**
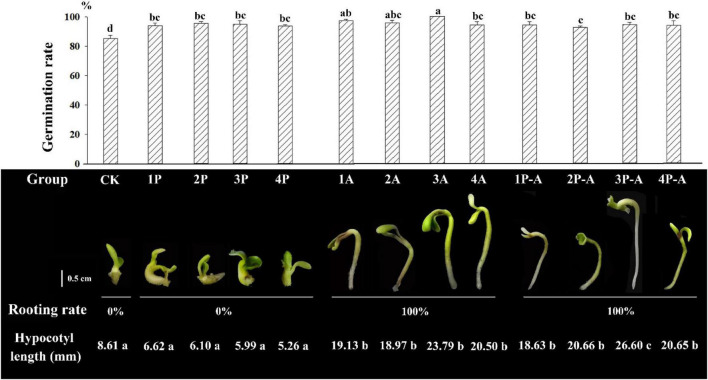
Germination rate, rooting rate, hypocotyl length, and seedling morphology of seed embryos treated with different anti-browning agents. CK represents the control group; 1–4P represents the addition of PVP anti-browning agent groups with concentrations of 1.5, 2.0, 2.5, and 3.0 g•L^–1^, respectively. 1–4A represents the addition of AC anti-browning agent with concentrations of 0.3, 0.6, 0.9, and 2.0 g•L^–1^, respectively. 1–4P-A represents the groups added with 1.5, 2.0, 2.5, and 3.0 g•L^–1^ PVP and 0.9 g•L^–1^ AC anti-browning agent combinations, respectively. Rooting rate (%) = (Number of rooting seedlings/Number of germinated seedlings) × 100%. Different lowercase letters represent significant difference at 0.01 level (Duncan, *P* = 0.01).

Figure 1 shows that the germination rate of seedlings after adding the anti-browning agent is significantly higher than that of the CK group. Among them, the germination rate of 3A group can reach 100%, but the seedlings of different treatment groups show different morphologies. The hypocotyls of the embryo seedlings of the CK group were not significantly elongated, the average elongation length was 8.61 mm, the cotyledons and hypocotyls were enlarged, the growth rate was relatively slow, and the rooting rate was 0%. For the group added with PVP anti-browning agent (1P, 2P, 3P, and 4P), the embryonic seedling morphology was similar to that of the control group, the growth of roots was inhibited, there was no significant difference in the average elongation length of hypocotyl between different PVP treatment concentration groups and the CK group, and they have no difference in their growth. For the group added with AC antibrowning agent (1A, 2A, 3A, and 4A), the hypocotyl elongation was obvious, the mean elongation length was significantly longer than the CK and PVP groups (as shown in Figure 1), the cotyledons were not enlarged and were flat and wide at all treatment concentrations, but the effect of promoting rooting was significant, and the rooting rate was 100%. Testing with 0.9 g/L AC showed an excellent performance of peony rooting rate and seedling growth overall. The hypocotyls of the group added with two anti-browning agents (1P-A, 2P-A, 3P-A, and 4PA) were elongated, similar to adding the AC group only, but the hypocotyls were thinner, the cotyledons were smaller, except for 3P-A, the hypocotyl length was not significantly different at other concentrations (Figure 1), and the rooting rate can also reach 100%, indicating that both PVP and AC can increase the seedling induction rate of seed embryo by reducing browning, but only AC has the effect of promoting embryonic seedling hypocotyl elongation and rooting.

### RNA-Seq Analysis and Data Acquisition

Four groups (CK, 2P, 3A, and 3P-A) with better germination rates and obvious rooting differences in different treatment groups (marked directly as CK, PVP, AC, AC, and PVP) were selected for the comparative analysis of transcriptome sequencing. The Read Num and Base Num produced by 12 samples are shown in Table 3, and the Q20 percentages were over 90%, and the GC (number of G/C bases as a percentage of the total number of bases) percentages were over 45%.

**TABLE 3:** Base information statistics.

Sample	Read Num	Base Num	Q20 (%)	GC (%)
CK-1	49,145,224	6,748,074,777	97.27	45.61
CK-2	45,773,602	6,305,053,688	97.29	46.36
CK-3	41,531,112	5,722,152,220	97.14	45.87
PVP-1	51,767,768	7,058,031,299	97.39	46.8
PVP-2	54,583,640	7,382,919,737	97.09	46.71
PVP-3	45,214,764	6,193,826,414	97.32	46.83
AC-1	43,164,922	5,875,079,356	97.44	46.24
AC-2	42,173,928	5,845,653,992	97.48	46.44
AC-3	42,522,386	5,873,716,564	97.39	46.52
AC-and-PVP–1	43,128,464	6,044,250,064	96.75	46.23
AC-and-PVP–2	38,896,362	5,403,657,085	97.24	47.2
AC-and-PVP–3	24,363,904	3,415,330,847	90.75	47.08

*Read Num represents the total number of reads; Base Num represents the total number of bases; Q20 represents the percentage of bases with a Phred value greater than 20 to the total bases; and GC represents the number of G/C bases as a percentage of the total number of bases.*

**FIGURE 2:**
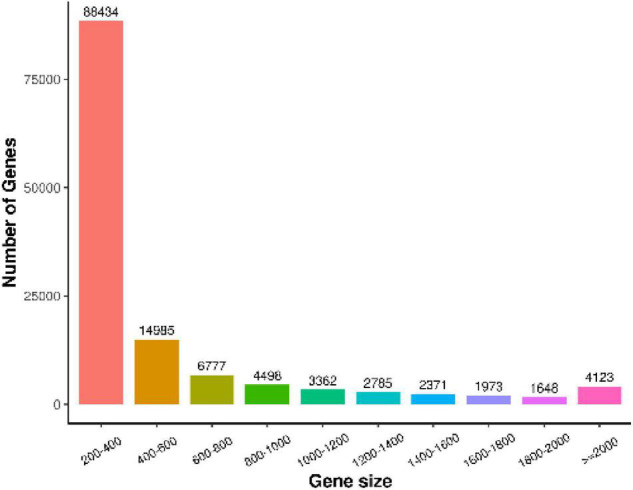
Length distribution statistics of assembly results. *X*-axis represents the range of gene length, and *Y*-axis represents the number of gene lengths within this range.

We used Trinity to assemble and select the longest transcript of each gene as a unigene (unigene is the only one obtained after de-redundancy and deduplication of all genes in each sample) and evaluated the assembly quality of all transcripts and unigenes (Table 4). All the sequences were assembled, and a total of 130,956 unigenes were generated. The statistics of the number of genes of different gene sizes are shown in Figure 2; the average length and median length were 515.16 and 293, respectively; the N50 was 724 nt.

**TABLE 4:** Genome assembly quality evaluation.

Index	All	Average length	Median length	Total assembled bases	N50
Transcript	199,689	588.17	337	117,450,803	898
Unigene	130,956	515.16	293	67,462,977	724

*N50 indicates that half of the assembled bases were incorporated into unigenes with a length of at least. All represents the total number of assembled transcripts and Unigene; average length represents the average gene size of the assembled transcript and Unigene; median length represents the median size of all assembled transcripts and Unigene; and total assembled bases represents the number of bases assembled in all transcripts and Unigene.*

Five authoritative databases of Swiss-Prot, PFAM, GO, KEGG, and eggNOG have been used to annotate these unigenes. Unigenes 22,483, 28,968, 29,101, 17,281, and 17,885 could be annotated to the eggNOG, GO, KEGG, PFAM, Swiss-Prot, and Swiss-Prot databases, respectively.

**FIGURE 3:**
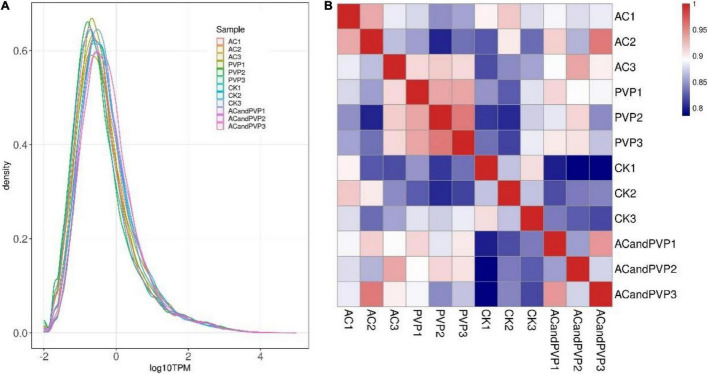
Gene expression abundance distribution map and sample correlation heat map. **(A)** The curves in different colors in the figure represent different samples, with the *X*-axis representing log10 (TPM) of the corresponding gene and the *Y*-axis representing probability density. Generally speaking, the number of differentially expressed genes (DEGs) only accounts for a small part of the whole gene, and a few DEGs have little influence on the distribution of the expression level of samples. In most cases, samples should have a similar distribution of expression levels; **(B)** the closer the correlation coefficient is to 1, the higher the similarity of expression patterns between samples, and the degree of correlation is positively correlated with the depth of color.

We standardized the sequencing depth and gene length and obtained the TPM value of the gene for subsequent analysis. Low-quality genes that were not expressed in more than 80% of the samples were deleted. The TPM values of qualified genes are shown in Supplementary Table 1. Different samples have similar gene expression distribution diagrams (Figure 3A). At the same time, the TPM expression of all genes in any two samples was used to analyze the correlation between biological replicate samples. The results showed that the Pearson correlation coefficients between the three biological replicate samples in each group were all greater than 0.8. It reveals that the three biological repeats are highly correlated and reproducible, which can be used for gene expression analysis (Figure 3B).

### Analysis of Differentially Expressed Genes Between Different Groups

**FIGURE 4:**
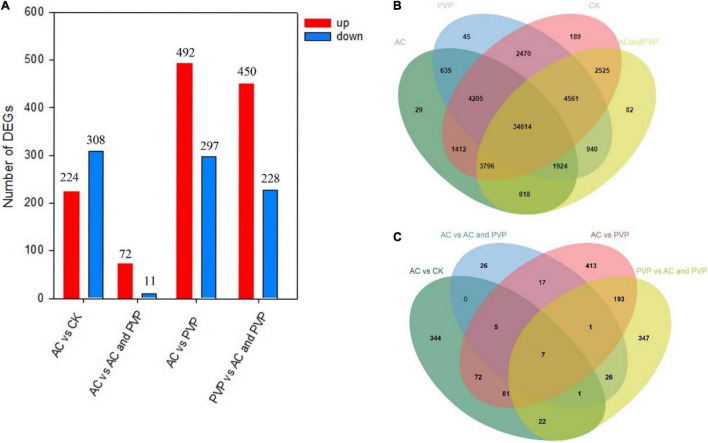
**(A)** The histograms of DEGs between samples; **(B)** Venn diagrams for transcriptome analysis of the expressed genes detected in the four groups; **(C)** Venn diagrams for transcriptome analysis of the DEGs detected between different groups.

We found that most of the genes were expressed in different groups, and the genes expressed in a group alone are relatively few compared with the total number of genes (Figure 4B), indicating that the addition of different anti-browning agents has little effect on the expression of the total number of genes in embryo seedlings. The number of DEGs between different groups is also relatively small compared with the total number of genes (Supplementary Tables 2–5), but when comparing between different groups, the number and expression of DEGs are significantly different (Figure 4A, the volcano map and heat map of DEG expression between different groups are shown in Supplementary Figures 1,2). Among them, the number of DEGs, whether upregulated or downregulated, between the two groups, AC vs. AC and PVP, is the least (Figure 4A). Moreover, the Venn diagram shows that the number of DEGs only expressed between these two groups is also the least (Figure 4C), indicating that the difference in gene expression between these two groups is small, and the difference between the AC vs. AC and PVP groups and the other comparison groups is that they both added AC.

### Gene Ontology Functional Classification and Kyoto Encyclopedia of Genes and Genomes Pathway Enrichment Analyses of Differentially Expressed Genes

**FIGURE 5:**
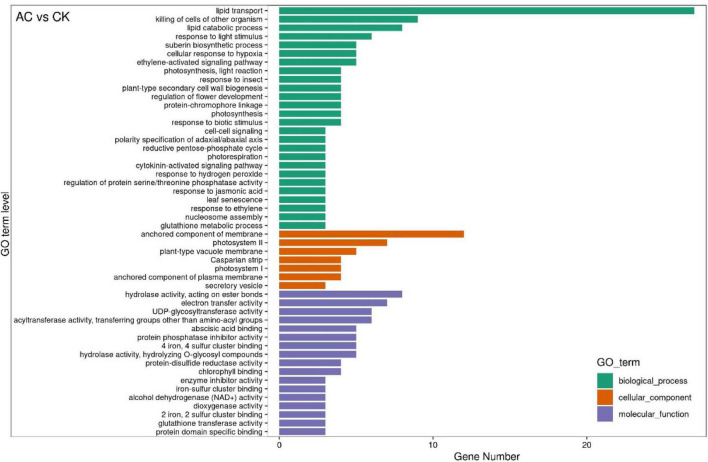
The GO functional classification of DEGs between the activated carbon (AC) and control check (CK). The graph is drawn using the first 20 terms with the smallest *P*-value. The ordinate is GO term, and the abscissa is the number of genes with different numbers of GO term.

The GO functional classification analysis shows that the DEGs between different groups were classified into 50 GO items, which were included in the biological process, the cellular component, and the molecular function, but the GO items compared between different groups were somewhat different [e.g., AC vs. CK group, Figure 5; the GO functional classification of other groups were shown in attached diagrams (Supplementary Figures 3–5)]. It is worth noting that the DEGs among the AC vs. CK, PVP vs. AC and PVP, and AC vs. PVP groups were mainly enriched in the “lipid transport” biological process (Figure 5 and Supplementary Figures 4,5), and the DEGs among the AC vs. AC and PVP groups were mainly enriched in “photosynthesis, light harvesting,” “protein-chromophore linkage,” “nucleosome assembly,” and so on (Supplementary Figure 3).

**FIGURE 6:**
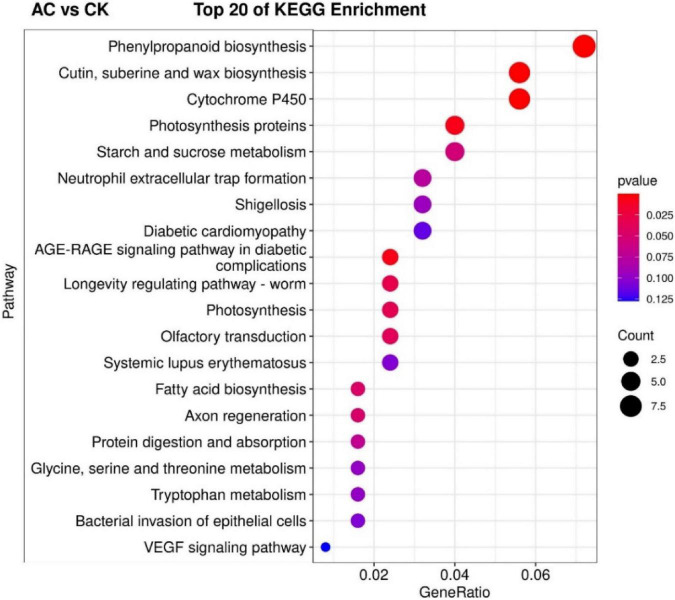
The top 20 of the Kyoto Encyclopedia of Genes and Genomes (KEGG) pathway enrichment of DEGs between the AC and CK. The first 20 pathways with the smallest *P-*values were used to draw the map, with pathway as the ordinate and enrichment factors as the abscissa (the number of differences in this pathway divided by all the numbers). The size of the circle indicated the number. The redder the color was, the smaller the *P*-value was, and the pathway representing the redder bubbles had more DEGs.

**FIGURE 7:**
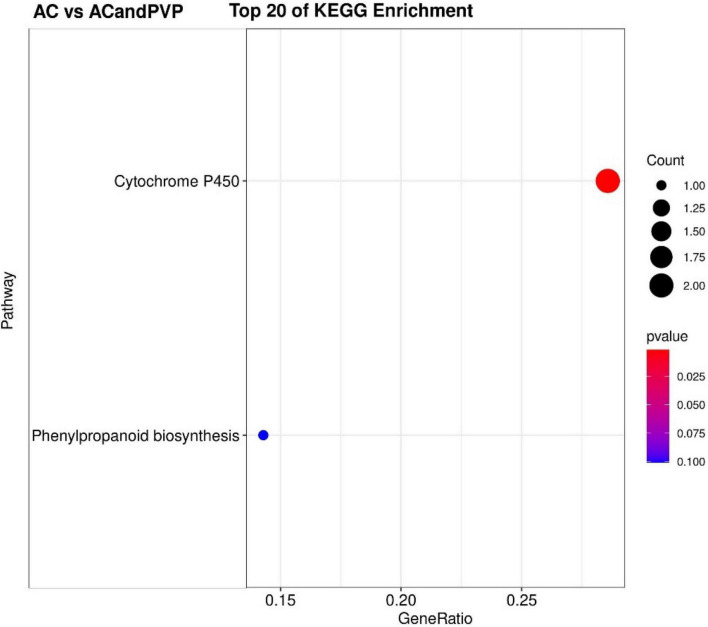
The top 20 of the KEGG pathway enrichment of DEGs in the group of AC vs. AC and polyvinyl pyrrolidone (PVP). The first 20 pathways with the smallest *P*-values were used to draw the map, with pathway as the ordinate and enrichment factors as the abscissa (the number of differences in this pathway divided by all the numbers). The size of the circle indicated the number. The redder the color was, the smaller the *P-*value was, and the pathway representing the redder the bubbles had more DEGs.

The KEGG pathway significant enrichment was used to determine the top 20 biochemical metabolic pathways and signal transduction pathways involved in the DEGs. The results were similar to GO functional classification. The metabolic pathways with the most significant enrichment of DEGs were the same among the AC vs. CK, PVP vs. AC and PVP, and AC vs. PVP groups, which were enriched in “phenylpropanoid biosynthesis” and “cutin, suberin, and wax biosynthesis” (Figure 6 and Supplementary Figures 6,7). There are only two metabolic pathways with significant DEG enrichment in the AC vs. AC and PVP groups, namely, “cytochrome P450” and “phenylpropanoid biosynthesis” (Figure 7).

### Real-Time Quantitative PCR to Verify the Expression of Differentially Expressed Genes

**FIGURE 8:**
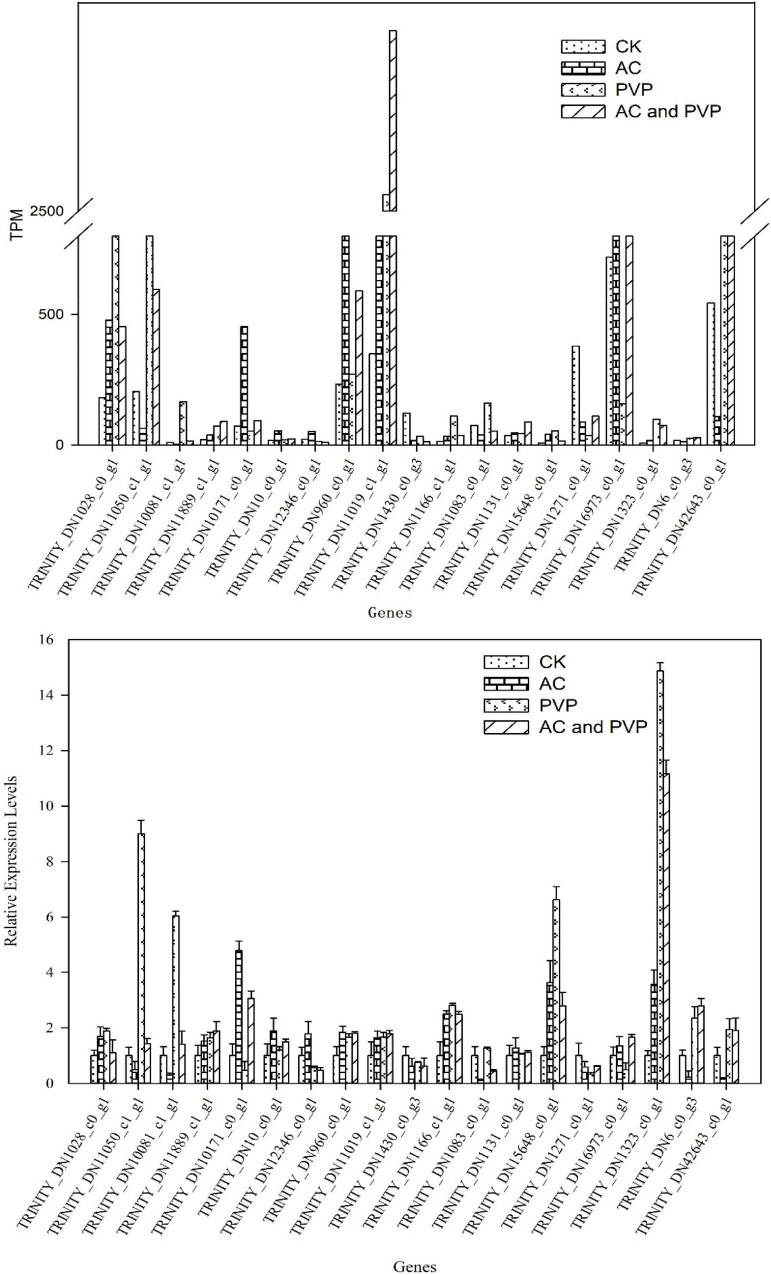
Transcripts per million expression value and qRT-PCR relative expression of DEGs.

To verify the accuracy of the RNA-seq results, 19 DEGs were randomly selected for qRT-PCR. The materials used for RNA extraction are the same as those used for transcriptome sequencing. The results showed that the relative expression trend of these genes in different templates was consistent with the gene expression trend of transcriptome sequencing (Figure 8), indicating that the transcriptome sequencing data are reliable.

## Discussion

Previous studies have mostly used plant hormones and culture components to analyze the effects of peony embryo tissue culture, but the mechanism of anti-browning agents on the rapid seedling formation of seed embryos is still unclear, and further research is needed (Liu et al., 2011). In this study, the effects of different anti-browning agents and their different combinations on the rapid seedling germination and rooting of the peony variety “FengDan” were analyzed. The results show that the addition of the two anti-browning agents, namely, AC and PVP, and their combination can effectively inhibit the browning phenomenon caused by damage during seed embryo peeling; compared with the CK group without adding any anti-browning agent, the germination rate of its seed embryos was significantly improved. In addition, the CK group and the PVP group can germinate but cannot take root at all. However, the AC and AC/PVP groups can not only increase the germination rate but also reach 100% rooting rate, and only the hypocotyls and roots of the plants in the AC/PVP group were thinner (Figure 1). It shows that both AC and PVP can prevent peony embryos from browning and increase the germination rate, while only the addition of AC and its combination with PVP can promote the further development of roots. To clarify its underlying mechanism, we conducted a transcriptome sequencing study.

Transcriptome sequencing data showed that there were 532,789, and 678 DEGs in the AC group compared with other groups (AC vs. CK; AC vs. PVP; AC and PVP vs. PVP), respectively; there were only 83 DEGs between the groups (AC vs. AC and PVP) that both have AC (Figures 4A,C). It shows that the gene expression of seed embryos has changed drastically after adding AC. These DEGs may be the key genes for AC to induce root development of peony seed embryos. At the same time, GO functional classification and KEGG pathway enrichment analysis showed that these DEGs were clustered in the same item between the AC vs. CK, AC vs. PVP, and AC and PVP vs. PVP groups, while the comparison between the AC and PVP vs. PVP groups was different from them (Figures 5–7). Among them, the DEGs of the AC vs. CK, AC vs. PVP, AC and PVP vs. PVP groups were mainly enriched in “phenylpropanoid biosynthesis” in the KEGG pathway enrichment analysis. The phenylpropanoid biosynthesis pathway has high physiological significance in plants, because it directly or indirectly produces all the substances in the phenylpropane skeleton, which plays a vital role in the growth and development of plants (Min et al., 2011; Liu et al., 2019). For example, one of the physiological functions of the important phenylalanine ammonia lyase (*PAL*) gene family in the phenylpropane metabolic pathway is to promote cell differentiation and plant growth (Yun et al., 2015). Through gene enrichment, phytohormone content determination, and metabonomic analysis, Wang et al. (2021) showed that the biosynthetic pathways of phenylpropanoids and flavonoids are related to root development, and flavonoid metabolism is accompanied by auxin biosynthesis and signal transduction to form the complex gene regulatory network in the process of main root development. Based on the results of this study, the gene families involved in this metabolic pathway are active, and most of them are upregulated, including peroxidase (*PER*), *PAL*, 4-coumarate: coenzyme A ligase (*4CL*), and caffeic acid 3-*O*-methyltransfease (*COMT*), especially the three peroxidase genes (*TRINITY_DN112784_c0_g1/TRINITY_DN649_c0_g1/TRINITY_DN9029_c0_g1*) among the three group comparisons showed upregulated expression (Supplementary Tables 2,4,5). It can be seen that AC can promote the development of roots by stimulating the expression of genes related to metabolic pathways of phenylpropanoid biosynthesis. The second enrichment pathway for these DEGs is “cutin, suberin, and wax biosynthesis.” Studies have shown that cuticle, lignin, and so on can provide mechanical strength for plants and limit water loss and pathogen invasion (Xin and Herburger, 2021), and drought resistance is also involved in a variety of genetic backgrounds (Ma J. et al., 2019), which to a certain extent shows that AC can promote peony embryos to quickly adapt to the medium environment, resist microbial pollution, and further promote rooting and development of seedlings; it is also one of the important reasons for thicker cotyledons and thicker hypocotyls in plants (Ursache et al., 2021). However, in the comparison between the AC vs. AC and PVP groups, the DEGs of KEGG pathway enrichment analysis are mainly enriched in “cytochrome P450,” and there are only two DEGs (*TRINITY_DN6_c0_g2/TRINITY_DN6_c0_g3*). Duan et al. (2017) found in the drought stress experiment of transgenic tobacco that overexpression of a spinach cytochrome p450 gene SoCYP85A1 can promote root development, enhance antioxidant enzyme activity, and regulate stress response. In our experiment, these two groups can promote rooting, but the root system development is obviously different. The roots of the group only added with AC is significantly stronger. It can be seen that these two DEGs are the key genes to promote the robust development of roots. It is worth mentioning that, in the comparison of the PVP vs. AC and PVP groups, only one gene (*TRINITY_DN112784_c0_g1*) was differentially expressed, and in the second enriched pathway “phenylpropanoid biosynthesis” of KEGG, it shows that, when both groups have AC, the effect of promoting root development through this metabolic pathway is minimal.

## Conclusion

Peony seed embryo-forming technology can make peony seed embryos to quickly become seedlings, to shorten the breeding cycle, and to accelerate the process of hybrid breeding. This study investigated the effects of different anti-browning agents and their combinations on the embryo and rooting of tree peony seeds through transcriptome sequencing, aiming to illustrate their potential regulatory mechanisms. In general, both AC and PVP anti-browning agents can inhibit browning and increase the germination rate. The effect is best when the AC concentration is 0.9 g/L, but only AC and the combination of AC and PVP can promote further rooting development. Transcriptome data show that it may be related to AC, which can stimulate the expression of genes related to phenylpropanoid metabolism to promote root development. At the same time, it enhances the tissue structure by strengthening the biosynthesis of the cutin and suberin, resisting microbial contamination, and promoting plant development. This study can provide a theoretical basis for perfecting the technology of rapid seedling formation of peony embryo tissue culture.

## Data Availability Statement

The data presented in the study are deposited in the GEO database of NCBI repository, accession number GSE192345.

## Author Contributions

XZ, XC, and CY planned and designed the research. CY, GX, and HZ participated in the experiment of seed embryo tissue culture. XC, WJ, and SJ were responsible for the mapping of transcriptome data. XZ and SJ provided advice on transcriptome data analysis. XC and XZ were involved in manuscript editing. JN and HY participated in the revision of the manuscript. All authors read and approved the final manuscript.

## Conflict of Interest

The authors declare that the research was conducted in the absence of any commercial or financial relationships that could be construed as a potential conflict of interest.

## Publisher’s Note

All claims expressed in this article are solely those of the authors and do not necessarily represent those of their affiliated organizations, or those of the publisher, the editors and the reviewers. Any product that may be evaluated in this article, or claim that may be made by its manufacturer, is not guaranteed or endorsed by the publisher.

## Footnotes

1
http://www.bioinformatics.babraham.ac.uk/projects/trim_galore/

2
http://eggnog-mapper.embl.de/

3
https://www.kegg.jp/ghostkoala/
