# Supplementary material for: The Effect of Anti-browning Agent Activated Carbon and Polyvinyl Pyrrolidone on the Rooting of Embryo Seedlings of “FengDan” and Its Transcriptome Analysis
Source: Front Plant Sci. 2022 Mar 21;13:832619. doi: 10.3389/fpls.2022.832619 (PMC8979295; doi:10.3389/fpls.2022.832619)
Supplement: Supplementary file 1 [file Data_Sheet_1.docx]

Supplementary Material

# Supplementary Figures

#
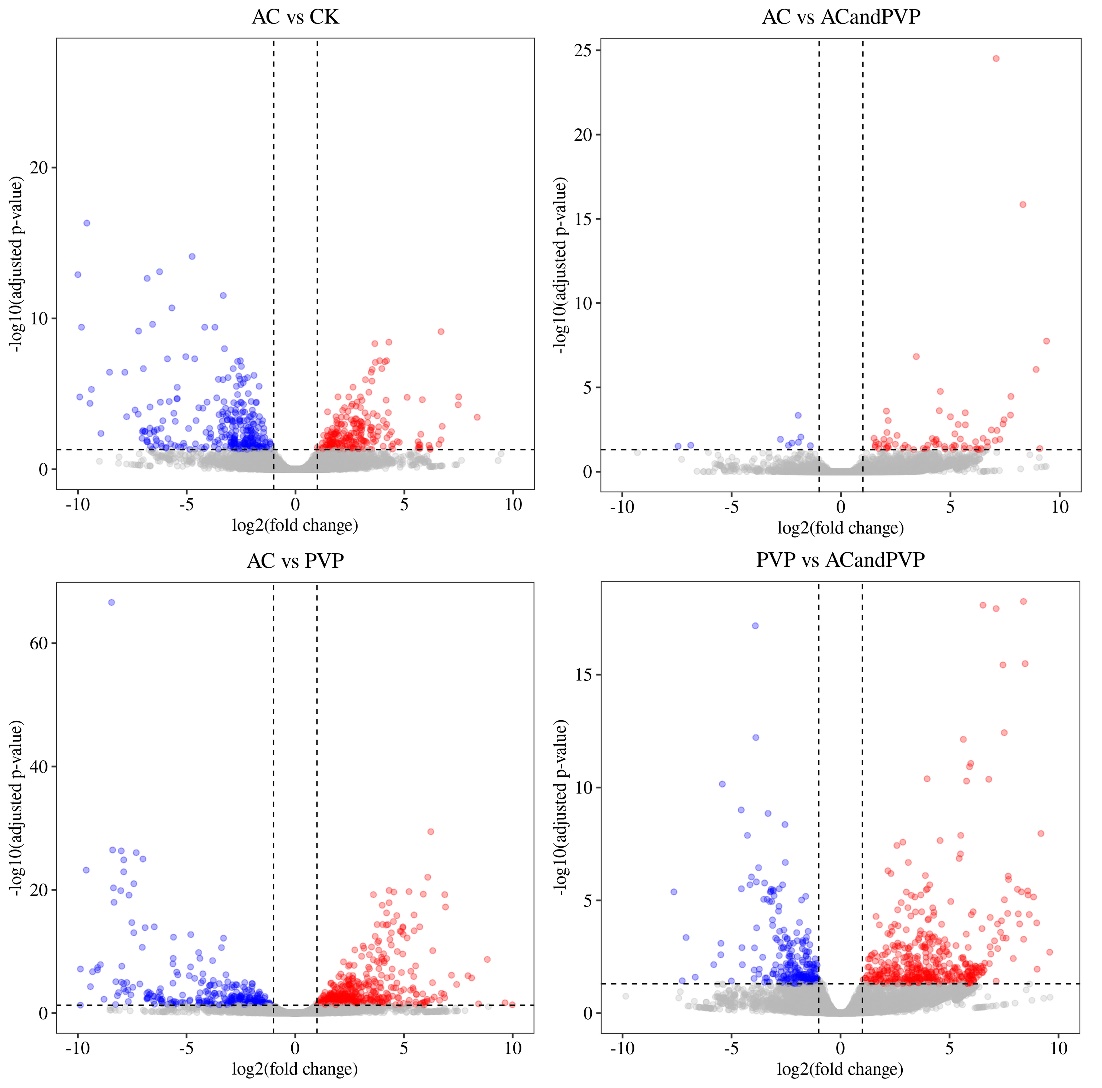


**Supplementary Figure S1.** Volcano map of DEGs comparison

X-axis in the volcano diagram: Log2 Fold Change, that is, the Log2 logarithm of the difference of expression levels between samples. Y-axis: Take negative logarithm (-log10) of the corrected P value; The closer the genes are to each end, the greater the difference; Red and blue represent up-regulated and down-regulated genes, respectively


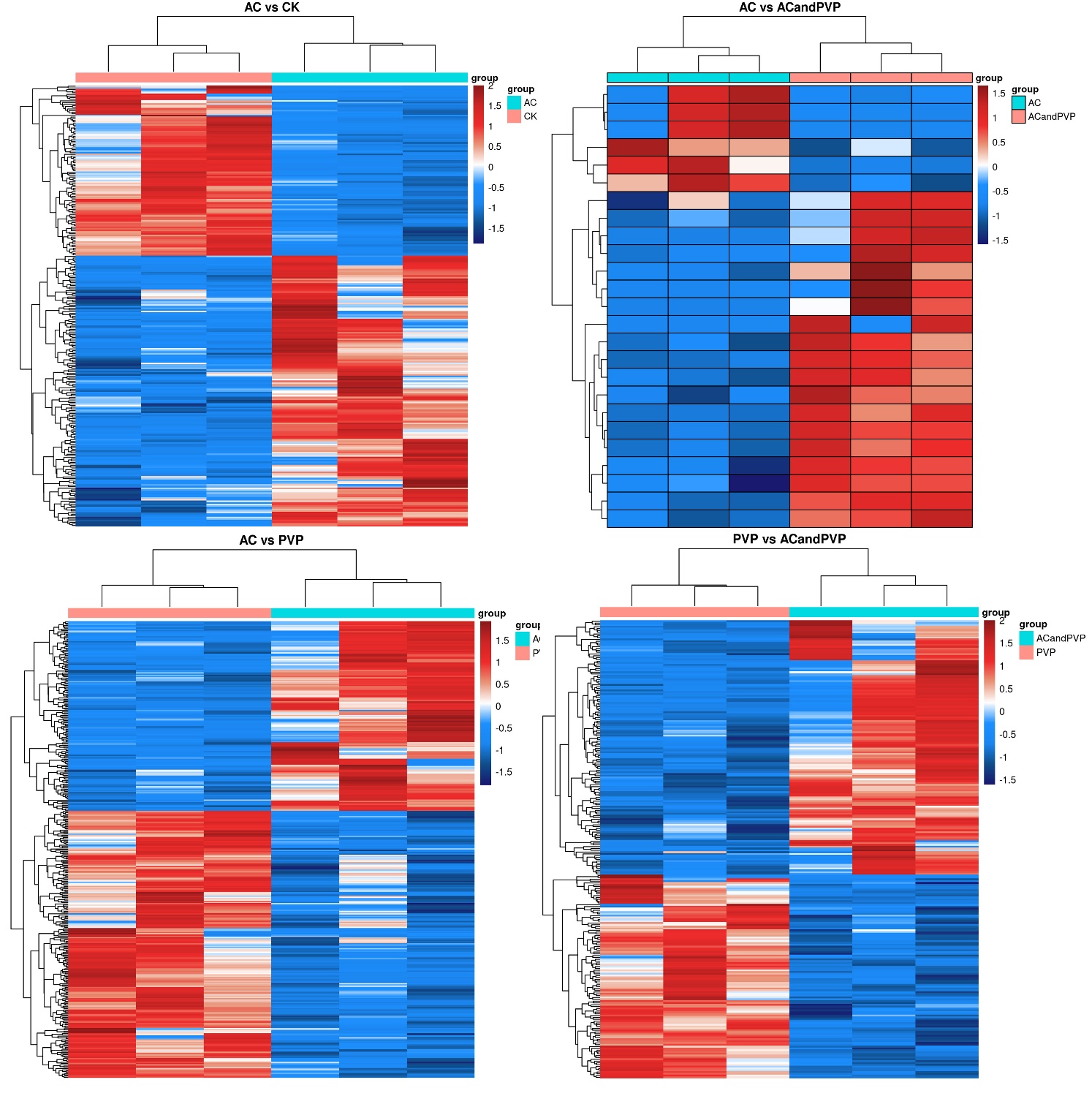


**Supplementary Figure S2.** Heat map of TPM of gene expression between different groups

The TPM heat maps of all differential genes in DESeq2 or edgeR analysis are shown in the figure. The red and blue scores represent the high and low TPM expression levels of this gene.


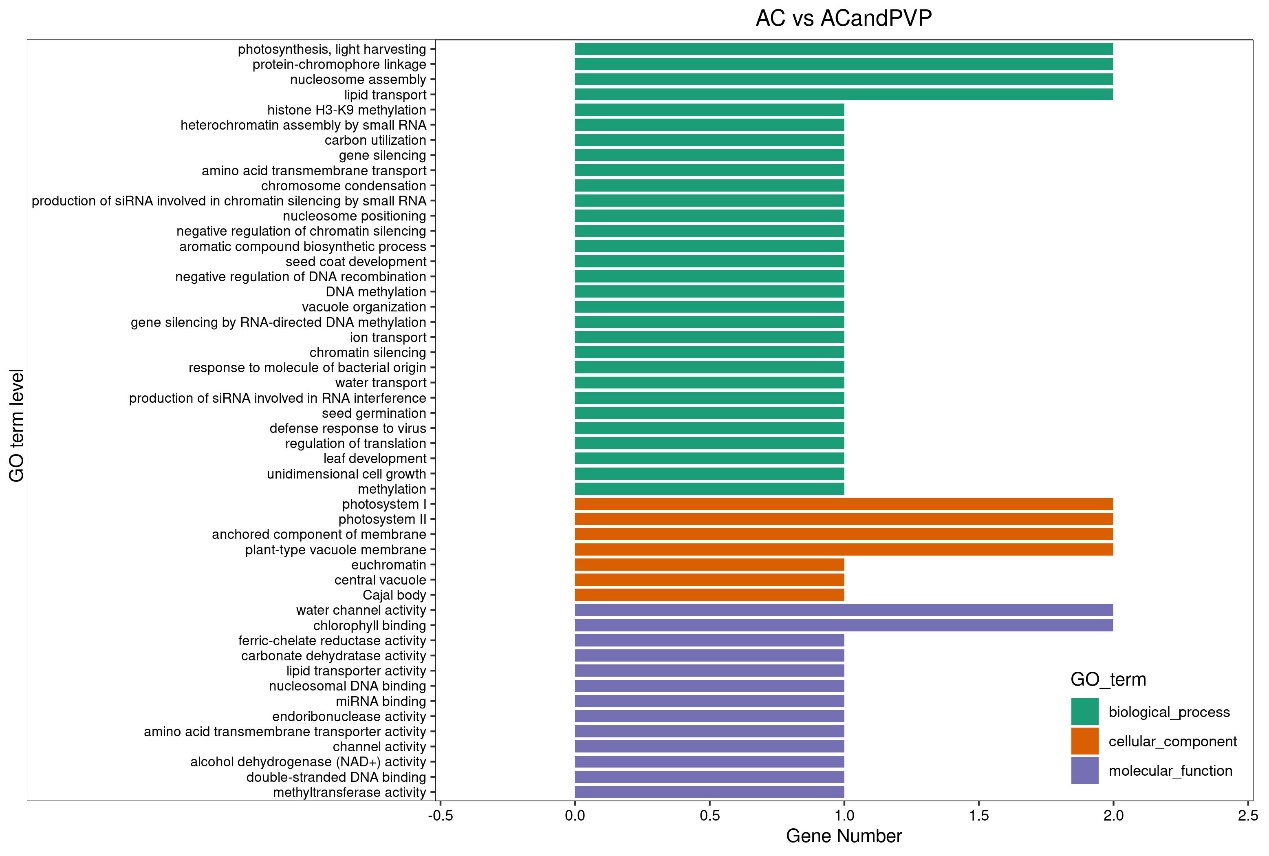


**Supplementary Figure S3.** GO functional classification of DEGs in AC vs AC and PVP

The graph is drawn using the first 20 terms with the smallest P value. The ordinate is GO term, and the abscissa is the number of genes with different number of GO term


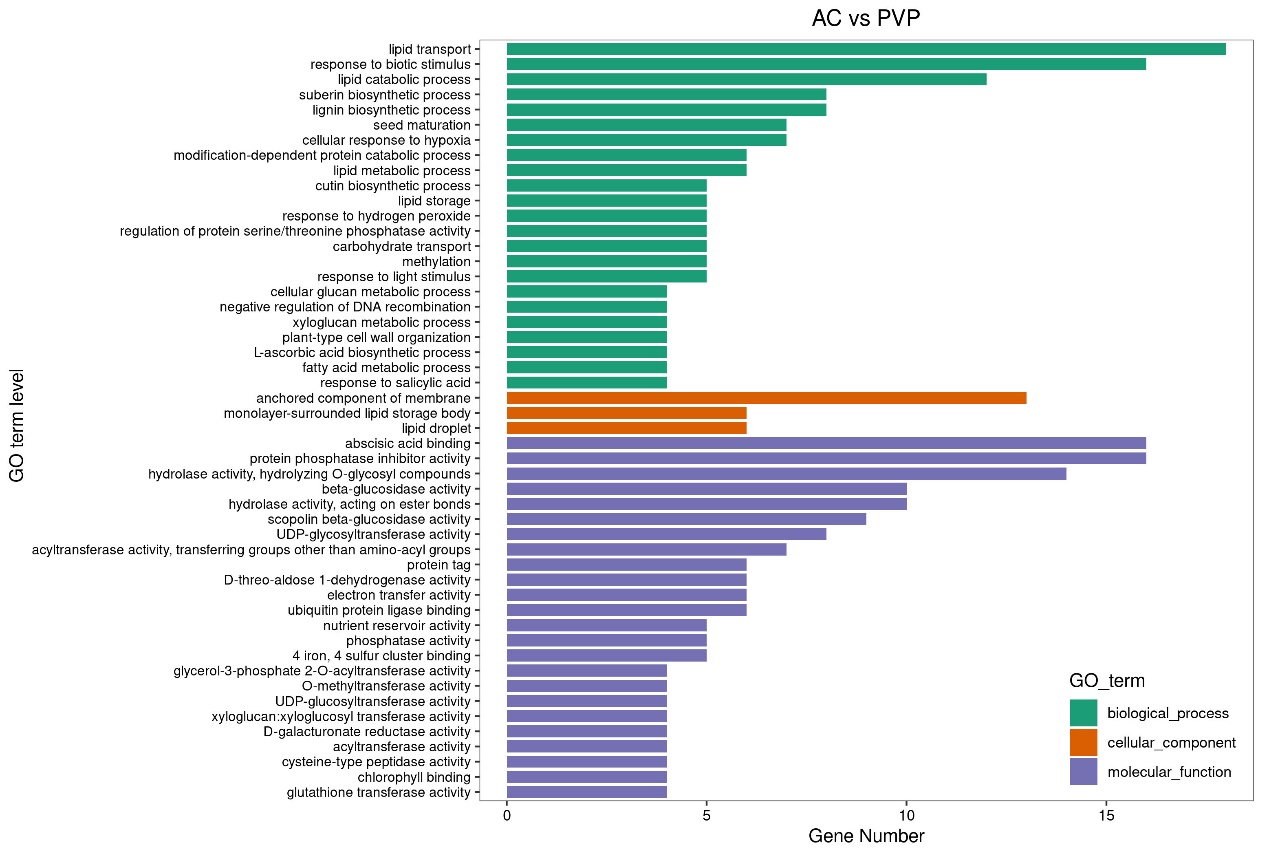


**Supplementary Figure S4.** GO functional classification of DEGs in AC vs PVP

The graph is drawn using the first 20 terms with the smallest P value. The ordinate is GO term, and the abscissa is the number of genes with different number of GO term


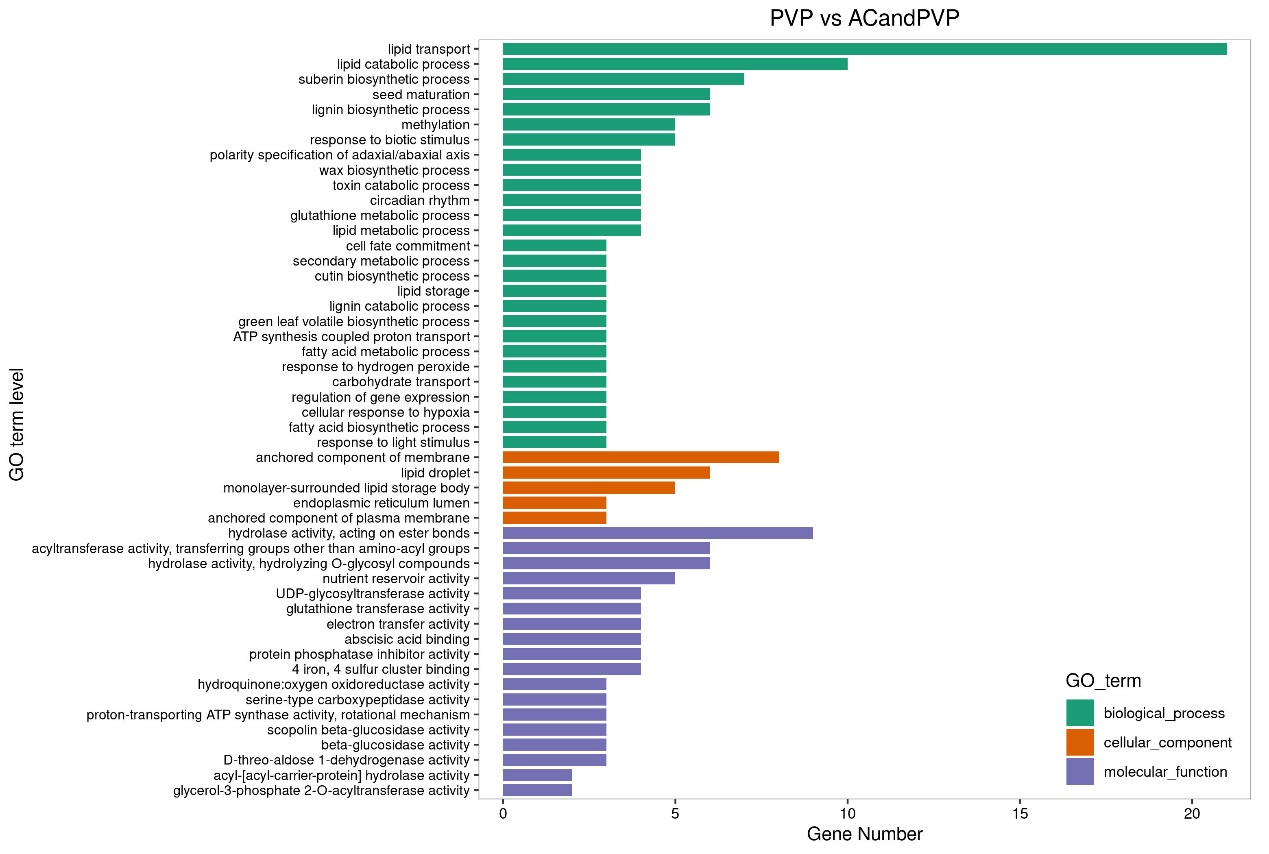


**Supplementary Figure S5.** GO functional classification of DEGs in PVP vs AC and PVP

The graph is drawn using the first 20 terms with the smallest P value. The ordinate is GO term, and the abscissa is the number of genes with different number of GO term


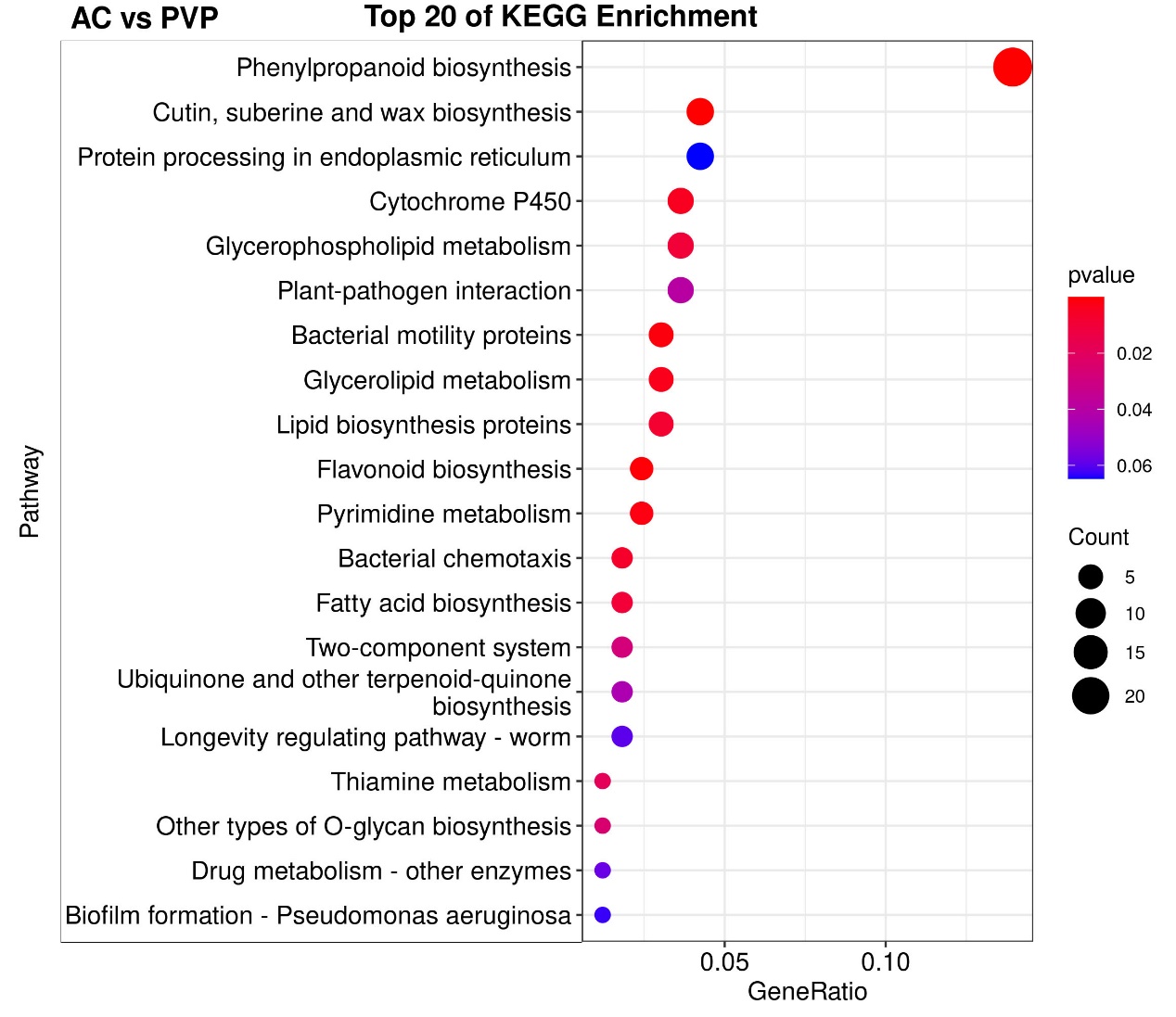


**Supplementary Figure S6.** The top 20 of the KEGG pathway enrichment of DEGs between the AC and PVP

The first 20 pathways with the smallest P values were used to draw the map, with pathway as the ordinate and enrichment factor as the abscissa (the number of differences in this pathway divided by all the numbers). The size of the circle indicated the number. The redder the color was, the smaller the P value was, and the pathway representing the redder the bubbles had more differential genes enriched


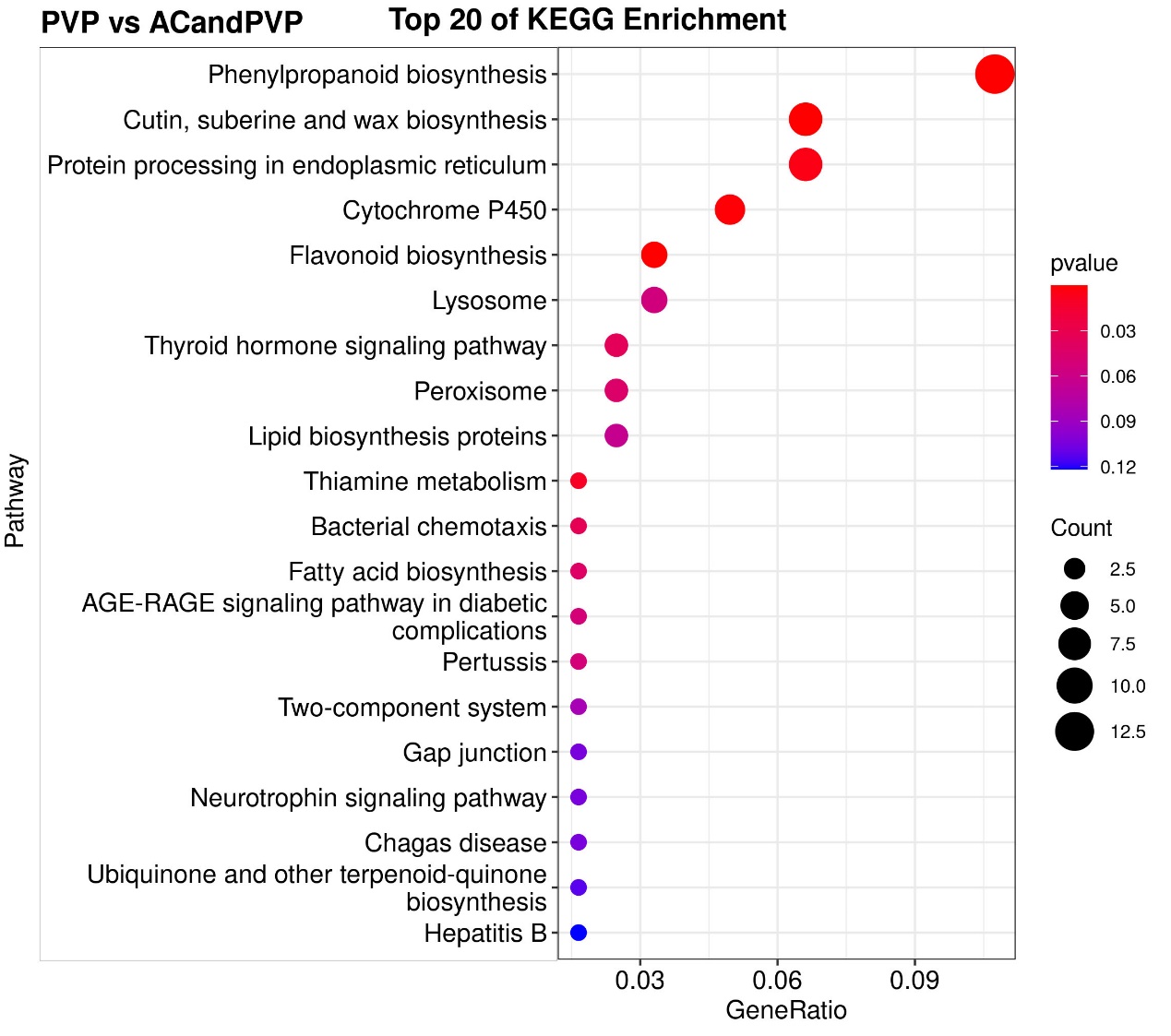


**Supplementary Figure S7.** The top 20 of the KEGG pathway enrichment of DEGs in the group of AC vs AC and PVP

The first 20 pathways with the smallest P values were used to draw the map, with pathway as the ordinate and enrichment factor as the abscissa (the number of differences in this pathway divided by all the numbers). The size of the circle indicated the number. The redder the color was, the smaller the P value was, and the pathway representing the redder the bubbles had more differential genes enriched
